# Implementation of a Web-Based Organ Donation Educational Intervention: Development and Use of a Refined Process Evaluation Model

**DOI:** 10.2196/jmir.8501

**Published:** 2017-11-30

**Authors:** Nakeva Redmond, Laura Harker, Yvan Bamps, Shauna St. Clair Flemming, Jennie P Perryman, Nancy J Thompson, Rachel E Patzer, Nancy S DeSousa Williams, Kimberly R Jacob Arriola

**Affiliations:** ^1^ Department of Behavioral Sciences and Health Education Rollins School of Public Health Emory University Atlanta, GA United States; ^2^ Georgia Budget and Policy Institute Atlanta, GA United States; ^3^ Department of Neurosurgery Emory University School of Medicine Emory University Atlanta, GA United States; ^4^ Emory Transplant Center Emory University Hospital Emory Healthcare Atlanta, GA United States; ^5^ Department of Epidemiology Rollins School of Public Health Emory University Atlanta, GA United States; ^6^ Department of Surgery Emory University School of Medicine Emory University Atlanta, GA United States

**Keywords:** Internet, intervention, evaluation methodology, program evaluation, research techniques, organ donation, health education

## Abstract

**Background:**

The lack of available organs is often considered to be the single greatest problem in transplantation today. Internet use is at an all-time high, creating an opportunity to increase public commitment to organ donation through the broad reach of Web-based behavioral interventions. Implementing Internet interventions, however, presents challenges including preventing fraudulent respondents and ensuring intervention uptake. Although Web-based organ donation interventions have increased in recent years, process evaluation models appropriate for Web-based interventions are lacking.

**Objective:**

The aim of this study was to describe a refined process evaluation model adapted for Web-based settings and used to assess the implementation of a Web-based intervention aimed to increase organ donation among African Americans.

**Methods:**

We used a randomized pretest-posttest control design to assess the effectiveness of the intervention website that addressed barriers to organ donation through corresponding videos. Eligible participants were African American adult residents of Georgia who were not registered on the state donor registry. Drawing from previously developed process evaluation constructs, we adapted reach (the extent to which individuals were found eligible, and participated in the study), recruitment (online recruitment mechanism), dose received (intervention uptake), and context (how the Web-based setting influenced study implementation) for Internet settings and used the adapted model to assess the implementation of our Web-based intervention.

**Results:**

With regard to reach, 1415 individuals completed the eligibility screener; 948 (67.00%) were determined eligible, of whom 918 (96.8%) completed the study. After eliminating duplicate entries (n=17), those who did not initiate the posttest (n=21) and those with an invalid ZIP code (n=108), 772 valid entries remained. Per the Internet protocol (IP) address analysis, only 23 of the 772 valid entries (3.0%) were within Georgia, and only 17 of those were considered unique entries and could be considered for analyses. With respect to recruitment, 517 of the 772 valid entries (67.0%) of participants were recruited from a Web recruiter. Regarding dose received, no videos from the intervention website were watched in their entirety, and the average viewing duration was 17 seconds over the minimum. With respect to context, context analysis provided us with valuable insights into factors in the Internet environment that may have affected study implementation. Although only active for a brief period of time, the Craigslist website advertisement may have contributed the largest volume of fraudulent responses.

**Conclusions:**

We determined fraud and low uptake to be serious threats to this study and further confirmed the importance of conducting a process evaluation to identify such threats. We suggest checking participants’ IP addresses before study initiation, selecting software that allows for automatic duplicate protection, and tightening minimum requirements for intervention uptake. Further research is needed to understand how process evaluation models can be used to monitor implementation of Web-based studies.

## Introduction

### Using Web-Based Behavioral Interventions

The lack of available organs is often considered to be the single greatest problem in transplantation today [1]. As of July 2017, there were more than 117,000 persons awaiting a life-saving organ in the United States; however, less than 28,000 deceased donor transplants were performed in 2016 [2].

Efforts to address the growing gap between organ supply and demand include an extensive list of face-to-face educational interventions, with the majority of these interventions designed to improve attitudes and knowledge related to organ donation and promote positive donation intentions [3-11]. The widespread use of the Internet, however, creates an opportunity to increase public commitment to organ and tissue donation through innovative Web-based educational interventions. Approximately 88% of US adults currently use the Internet, with all demographic groups showing an exponential increase in use in recent years [12]. Internet-based interventions within the context of behavioral health research have capitalized on the high accessibility of the Internet and proliferated in the past 20 years [13], particularly in topics such as human immunodeficiency virus [14-16], depression and anxiety [17,18], and eating disorders [19,20], whereas Internet-based interventions addressing organ donation have only minimally been explored. Despite the pervasiveness of Web-based interventions, process evaluation frameworks able to assess their implementation are lacking.

Web-based interventions offer several advantages including fast and easy participant recruitment and data collection [21-27] and minimal cost compared with in-person interventions [15]. Internet interventions have been shown to have high fidelity, are transferrable to a variety of settings, and are scalable [15]. The added anonymity of the Internet reduces potential issues of social desirability and stigma, which is particularly appealing for studies addressing sensitive health topics. In addition to significantly improving knowledge, attitudes, and associated behaviors for various health topics, there is some evidence that compared with the in-person versions of the intervention, Web-based versions may be more effective [17,20,28]. Web-based organ donation initiatives have been successful in improving organ donation–related knowledge [29], attitudes [29,30], and behaviors [30], and reaching minority populations [31] who are consistently underrepresented as donors [2].

### Assessing Fraud in Internet Studies

Despite these benefits, Internet studies present a list of disadvantages including high levels of attrition [32] and inability to achieve a representative sample [33]. Anonymity on the Internet can be considered a strength, but it also adds the potential of fraudulent and repeat responders [34]. Fraud in Internet studies has previously been cited as rare [35] and not compromising to the overall quality of data collected in Web-based studies [34]. However, it might be that investigators underestimate the extent of fraudulent responses and their impact on the internal validity of the associated studies. Subject fraud often involves elaborate adaptive strategies to counter detection measures in place and run virtually undetected even during data analysis. In one sexual behavioral risk Web-based study, nearly 11% of all responses were deemed ineligible because of fraud or repeated entry, with one respondent responsible for 6% of all responses [36]. Similarly, in a Web-based study surveying men who have sex with men, 11.6% of all entries were determined to be fraudulent and a result of changing responses on the study screener to gain entry or repeated entry into the study [37]. Further analysis demonstrated that including these fraudulent data would have greatly skewed study findings [37], underscoring the threat of fraudulent responders.

Few studies have offered explicit methods for preventing fraud from occurring in Web-based studies. Suggestions occur at differing stages of the research process and range from simple strategies such as eyeing suspicious entries (such as designated birthday does not match self-reported age) [35], asking participants whether they have already participated in the study before initiating [34,38], or flagging repeated emails and usernames [35,37-39], to more complex approaches such as altering study design (ie, requiring a telephone interview before participation) [35,36] or monitoring participants’ Internet protocol (IP) addresses to determine participants’ geographic locations and repeat responders [35]. Despite the range of options to prevent fraud, all methods have been found to be problematic [35,38] and sometimes labor intensive, with even the more advanced fraud detection methods requiring some level of manual checking [35,36]. The emerging use of “bots” or automated devices designed to hack studies without human effort, present an additional, more complex obstacle for preventing fraud and protecting study data. And although CAPTCHA (Completely Automated Public Turing test to tell Computers and Humans Apart) is used to prevent “bots” from accessing Internet forms (usually prompting users to type in the name of an image or check a box), this function is not always successful in blocking these fraudulent attempts and may unintentionally deter valid participants with low computer literacy [35]. The ever-changing landscape of the Internet, and consequently Web-based interventions, requires equally dynamic security measures [35] and an understanding that confirming eligibility among all participants may not be possible in Web-based studies [36].

### Assessing Intervention Uptake in Internet Studies

An additional concern in implementing Web-based studies is ensuring uptake of the intervention. Most Web-based study participants have the convenience of completing the study on devices in their own homes and on their own time. Whereas such autonomy within the context of a research study may alleviate accessibility issues among the target population [21], it also limits implementer control and presents the risk of insufficient uptake of the intervention. Overall, measured intervention uptake on Web-based platforms has been found to be less than intended [40], as participants have been found to abandon the intervention before finishing [40] and “clicking through” associated questionnaires without reading all of the content [41].

Ensuring uptake of Web-based interventions promoting organ donation is especially challenging. Organ donation registration has been characterized as a low-vested-interest health behavior that is accompanied by high attitudinal ambivalence and a lack of personal benefit [42], thus making it difficult to appeal to the general population. Moreover, there is evidence that distrust of the medical care system and physicians undergird negative attitudes about organ and tissue donation, particularly among African Americans [43]. These conditions may make uptake of a Web-based organ donation education intervention particularly challenging within this population.

Although Web-based studies are more difficult to control, Web-based analytics allow study implementers to unobtrusively monitor participants’ engagement with the study [41], with log-in rates, website hits (number of people who landed on the website), pages visited, modules completed, and completion of the entire intervention serving as the most commonly used exposure measures [40]. Monitoring uptake is not only becoming more common but expected for Internet studies [21], creating a dual opportunity to assess participant engagement and potentially identify fraud [44] by monitoring patterns of website use.

Measuring uptake of in-person intervention materials is common practice and helps attribute specific changes in study outcomes to varying levels of exposure to the intervention [45]. Measuring uptake for Web-based studies, however, is less explored, and has yielded mixed results in terms of project outcomes. For example, Christensen et al [46] measured intervention uptake of a Web-based cognitive behavioral therapy study and found that participants’ anxiety and depression scores significantly lowered with each additional module completed and more time spent on the modules. In a Web-based smokeless tobacco cessation program, Danaher and Seeley [21] found Web-based program exposure to mediate the relationship between self-efficacy and tobacco abstinence. And although it may be expected that increased exposure to intervention materials is positively associated with outcomes, Glasgow et al [32] found that ongoing engagement was negatively associated with the amount of material presented in the intervention, which of course presents a potentially new threat to the study design and to desired outcomes.

### Evaluating Implementation of Internet-Based Interventions

Some efforts have been made to determine how to implement Internet interventions in ways that maximize their effectiveness. For example, Cummins et al (2003) created the 5 As (advise, assess, assist, anticipatory guidance, and arrange follow-up) for effective health behavior change treatment on the Internet framework to address an emerging need for evaluation frameworks for Internet studies [47]. However, this model is limiting as it was adapted for preventive behaviors and disease management mostly focused on assessing content and admittedly only “provided the minimum criteria for a program to have the potential for providing behavior change” [47]. More recently, O’Grady et al (2009) created a dynamic evaluation framework for interactive Web technologies [48]. Although this framework is extensive, it maintains a conceptual approach and leaves the question of how to practically implement such a robust evaluation model, which is an overall critique of Web-based evaluation models [49]. Additional evaluation efforts on the Internet have been designed for static websites that provide health information and not interactive websites [50] and have mostly relied on self-report to assess usability [51], quality [51], and uptake [21,40,52,53] of the website. Whereas these evaluative techniques are insightful, more rigorous and objective measures are necessary for implementers to fully explore implementation of interactive Web-based interventions. Process evaluation is necessary to explain results according to intervention component, assess quality, and accuracy [45] and to help explain why a program was or was not successful [54]. Conducting a process evaluation to understand how Internet studies are implemented is imperative to optimize the Web-based platform, ensure program uptake, minimize waste, and maximize intervention effectiveness.

The purpose of this study was to investigate how a culturally sensitive, Web-based intervention aimed to increase organ donation among African Americans in Georgia was implemented by using a process evaluation model adapted for Web-based interventions. Drawing from previously developed process evaluation constructs [45], we adapted reach, recruitment, dose received, and context for use in Internet-settings. We excluded dose delivered and implementation from our adapted model, as all intervention components were delivered on a standardized Web-based platform. We also did not formally include fidelity (an original construct measuring the overall quality of intervention implementation) in our process analyses because of its strong relationship with the included process evaluation constructs and inherent measurement challenges [45]. Relevant to each process evaluation construct, this study sought to answer six research questions:

1. Reach: What proportion of interested participants was found eligible for the study?
2. Reach: What proportion of eligible participants participated in the study?
3. Reach: To what extent were Georgia residents recruited into the study?
4. Recruitment: To what extent were Web recruiters successful in attracting participants to the study?
5. Dose received: To what extent did participants engage in the study intervention components?
6. Context: To what extent did the Web-based setting influence study implementation?

## Methods

### Study Design and Sample

Project WEB ACTS (About Choices in Transplantation and Sharing, ACTS) is a Web-based intervention designed to assess the feasibility of using the Internet to deliver culturally sensitive, educational materials about organ donation and to increase donor registration among African American adults in Georgia. We used a randomized controlled pretest-posttest design to compare the effectiveness of the intervention website (Project WEB ACTS) to the control website (Donate Life Georgia). The Donate Life Georgia website features general facts about organ and tissue donation and personal donation stories, both nonspecific to any particular racial group. There is also a link to sign up on the Georgia state donor registry on the Donate Life website. Eligible participants identified as African American or black, were residents of Georgia, were 18 years of age or older, and were not already registered on the Georgia state donor registry, as indicated by self-report. This study was reviewed by the Emory University Institutional Review Board and considered exempt from further review and approval (IRB00078995).

### Website Design

**Figure 1:**
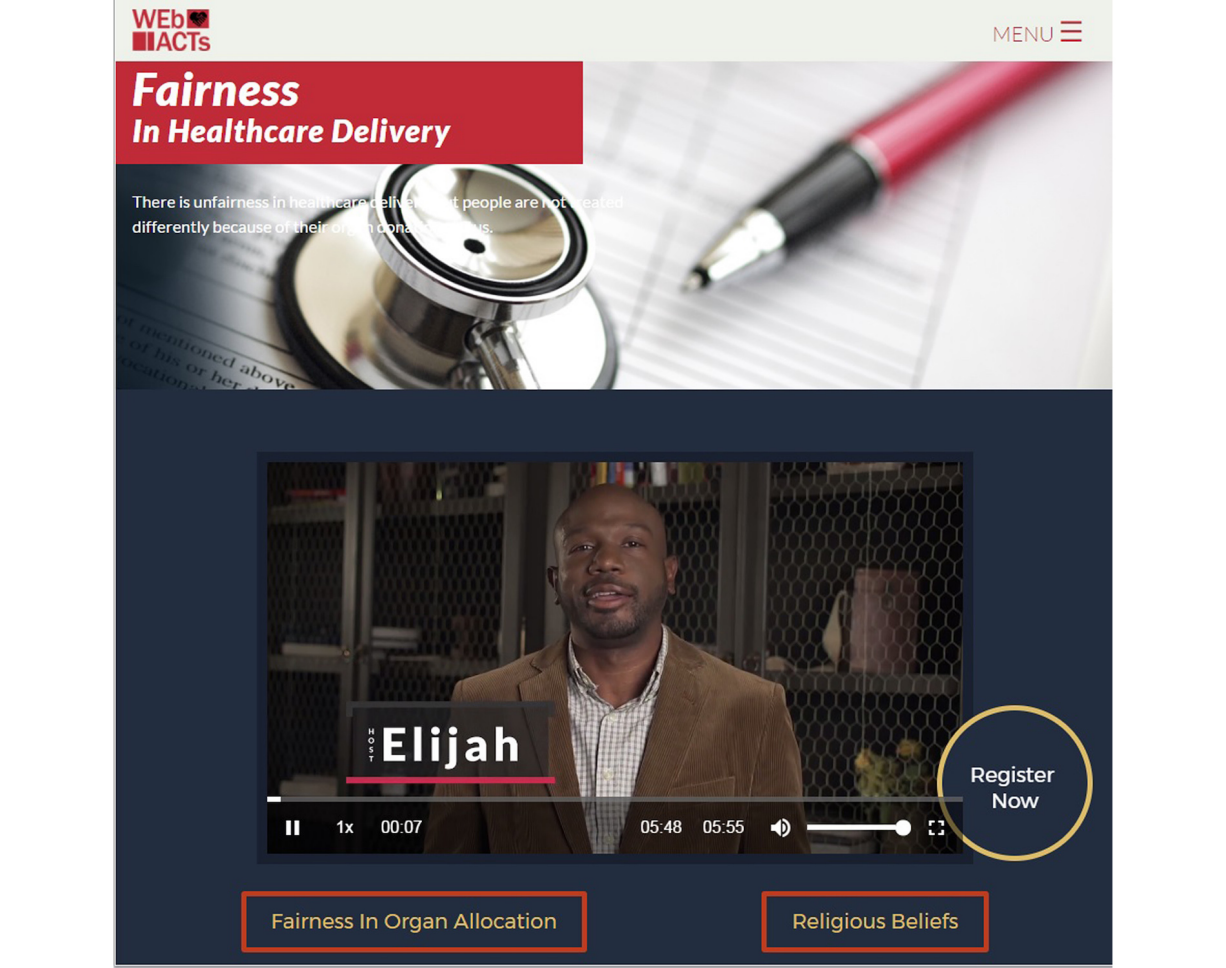
Intervention (Project WEB ACTS) interactive website, Fairness In Healthcare Delivery module.

Using organ donation literature, the two-dimensional model of cultural sensitivity [55], and the IIFF model of donor registration [42], we identified five prominent organ donation topic areas relevant for African Americans [56,57]. The Project WEB ACTS website modules are Act Now, Fairness in Organ Allocation, Fairness in Health Care Delivery, Religious Beliefs, and Let’s Talk About Life. Each module contains a corresponding 3- to 4-min video featuring an African American host and individuals discussing the importance of organ donation from various perspectives (ie, pastors, physicians, and living donors). The final tab of the website features a link leading to the Georgia state donor registry for those participants who decide to register as an organ donor at the conclusion of the intervention. A website consulting company was hired to develop the Project WEB ACTS website. All content was adapted from prior iterations of Project ACTS [58-60] and informed by both formative and scientific research (Figure 1).

### Data Collection Procedures

We identified and trained 17 Web recruiters to recruit participants into the study, primarily using Web-based mechanisms (eg, social media, Web-based listservs, and electronic bulletin boards). Web recruiters were demographically similar to the target population, familiar with Internet technology, and maintained strong online networks. Using approved language, Web recruiters advertised the Project WEB ACTS study, with each posting including a brief description of the project, mention of the incentive (US $10 virtual gift card), and a URL link to the study eligibility screener. The participant incentive was considered appropriate for the anticipated time burden of the proposed intervention according to prior Web-based studies [32,36], yet was kept nominal to reduce the risk of repeat participants [39]. Convenience sampling was used to identify eligible participants. There was a strong emphasis on online recruitment; however, recruiters were also permitted to share paper recruiting materials, including hard copy flyers with their in-person social networks (eg, at church, work, or in their neighborhood). Additionally, Project WEB ACTS administrators briefly (for approximately 24 hours) advertised the study on the Craigslist website to recruit participants into the study.

The project coordinator determined individuals’ eligibility based on answers to a Web-based eligibility screener and informed participants of their eligibility status via email. Emails to eligible participants included a link leading to the study consent and baseline assessment (a 38-item questionnaire measuring attitudes and beliefs regarding donation and transplantation, knowledge of the donation and transplantation system, Internet usage, and demographic characteristics). After completing the baseline assessment, participants were randomized to either the intervention or control website and required to spend a minimum of 10 min on the site before the posttest questionnaire was made available. The posttest questionnaire consisted of 24 items, including the attitudes, beliefs, and knowledge measures from the pretest and an added two items measuring the likelihood that the respondent would register as an organ donor in the future. Both intervention and control websites included a “call to action” for donation registration, and participants were allowed to sign up as organ donors on the Georgia state donor registry while on either website. Control and intervention participants were provided with a code upon completion of the posttest questionnaire and instructed to email the project coordinator with the code to receive the incentive. Each email address was checked before distributing the virtual gift card to ensure that multiple incentives were not sent to the same email address.

### Adaptation and Implementation of Process Measures

We first analyzed frequencies of all usable data using Statistical Package for the Social Sciences (SPSS) version 23.0 (IBM Corp). Drawing from previously developed process evaluation constructs [45,54,61], we adapted reach, recruitment, dose received, and context for Internet-based interventions and used each to conduct a process evaluation of our study (Table 1).

#### Reach

Reach is most commonly considered the proportion of the target audience that participates in the study [45,54]. We can estimate our target audience (African American adults living in Georgia and not registered as organ donors) given census data and state registry data; however, those individuals who visited the study eligibility site better represent our target, given the absence of any widespread, population-level marketing. The breadth of the Internet and our use of convenience sampling through online networks make it difficult to determine a numeric value of the intended target audience, requiring the adaptation of the reach construct. As a result, we defined reach as the proportion of interested participants who were found eligible for the study and the proportion of eligible participants who participated in the study. The study’s project coordinator manually checked each participant’s eligibility screener before study entry.

We also defined reach as the extent to which Georgia residents were recruited into the study. Although the Project WEB ACTS study was administered entirely on the Web, it was important to assess the physical environment of participants, as this study sought to explicitly recruit Georgia residents to register for the Georgia state donor registry. States maintain separate donor registries; therefore, including participants outside of the state of Georgia would undermine our ability to document registration on the Georgia state donor registry. We used IP addresses to approximate each participant’s location at the time of the study and to assess the extent to which Georgia residents were recruited into the study. First, we retrospectively analyzed each participant’s IP address to determine his or her approximate location at the time of completing the study and to identify repeated IP addresses. Similar to a street address, an IP address is a unique set of numbers used to identify computers on a network [39]. Whereas multiple individuals who share a home or work in the same building may be able to use one IP address, we expected such instances to be minimal. We used a reputable Internet IP address search engine to obtain IP information and used a second recommended IP address search engine to spot check results from the first search engine. IP addresses were used to estimate participant location (United States vs non-United States and US state) and to identify study repeaters.

**Table 1:** Adapted process measures applied to Project WEB ACTS (About Choices in Transplantation and Sharing) intervention.

Construct	Definition	Research question	Indicators
Reach	The extent to which individuals are found eligible, and participate in the study	What proportion of interested participants was eligible for the study?	Percentage of individuals who completed the eligibility questionnaire and were found eligible for the study
		What proportion of eligible participants participated in the study?	Percentage of individuals who were found eligible per the eligibility questionnaire and completed the study
		To what extent were Georgia residents recruited into the study?	Location estimation (Georgia vs other US state) per retrospective Internet protocol address analysis
Recruitment	Procedures used to approach and attract participants	To what extent were Web recruiters successful in attracting participants to the study?	Percentage of participants referred to study from Web recruiters and other methods
Dose received	The extent to which participants actively engage with, interact with, are receptive to, or use materials or recommended resources	To what extent did participants engage in the study intervention components?	Number of website sessions Percentage of new sessions Number of users Average time spent on the entire website (min:sec) Number of page views Average time on each page (min:sec) Number of individuals who pressed “play” for each video Highest percentage completed of each video Number of individuals completing the corresponding highest percent completed
Context	Aspects of the Web-based setting that may influence intervention implementation	To what extent did the Web-based setting influence intervention implementation?	Data trends of eligibility screener responses as compared with Web-based marketing efforts Debriefing interviews with Web recruiters

Respondents with IP addresses outside of the United States, IP addresses outside of the state of Georgia, and chronically repeating IP addresses (defined as those repeating more than 3 times) were identified as having concerning IP addresses. All concerning entries were flagged for further consideration.

#### Recruitment

Participants were asked to designate how they were recruited into the study, with answer choices including Web recruiters (listed by name) or other methods (which we inferred to be our Craigslist website advertisement). Given the emphasis on recruitment through social media and Internet forums and the difficulty in objectively tracking recruitment, recruitment source was measured by self-report.

#### Dose Received

We used Google analytics software to assess dose received or the extent to which participants were exposed to the WEB ACTS intervention website. This software allowed us to collect aggregate data on uptake of the website, including number of users, number of page views, average time spent on each page, and highest percentage completed of each of the five videos. Participant usage data were not available for control participants accessing the Donate Life Georgia website because it is a public webpage.

#### Context

Context in traditional study settings considers the “larger physical, social, and political environment that either directly or indirectly affects an intervention” [45]. The Internet is its own environment, with its own culture [44]; thus, context may be assessed as the Web-based setting to determine what factors in the virtual study environment may have affected intervention implementation. The dynamic nature of the Internet, however, makes it difficult to monitor activity broadly. We focused on Web-based environments where the study was advertised, including social media pages and Craigslist website, with the understanding that assessing where the study link could be accessed could provide information regarding most effective recruitment sites, sites potentially encouraging fraud, and other contextual factors affecting overall study implementation. We used data trends as a proxy indicator for assessing context, including responses to the eligibility screener as compared with Web-based marketing efforts and also conducted debriefing interviews with Web recruiters to gain insights to events happening on social media sites used for recruitment and to corroborate our data.

## Results

### Participant Demographics

Project WEB ACTS opened for recruitment on September 21, 2015 and closed on November 13, 2015, and a total of 772 participants were included in our initial participant pool. The majority of participants were male (56.2%, 434/772), and ages ranged from 18 to 74 years with a median age of 37 years. Most of the participants were married (74.8%, 577/772). Approximately half of all the participants reported college as the highest level of education completed, and the vast majority of participants were working full-time or part-time (94.3%, 728/772; Table 2).

### Reach

A total of 1415 individuals completed the eligibility screener; 948 (67.00%, 948/1415) were determined to be eligible based upon their responses to this screener. Of the 467 (33.00%, 467/1415) ineligible participants, 109 were not African American, 6 were not residents of Georgia, and 449 indicated that they were already on the Georgia state donor registry (criteria were not mutually exclusive). Of the 948 eligible participants, 918 (96.8%) completed the study. However, after eliminating duplicate entries (those with repeat emails or usernames; n=17), those who did not initiate the posttest (n=21) and those who provided an invalid ZIP code (n=108), we ended with a total of 772 surveys to use for further analysis.

Per the IP address poststudy analysis, 600 (77.7%, 600/772) respondents had an IP address within the United States, whereas 172 (22. 3%, 172/772) had an IP address outside of the United States. Of those in the United States, 577 (96.2%, 577/600) had IP addresses outside of Georgia, and 23 (3.8%) had IP addresses within Georgia. Of the 23 respondents who completed the study within Georgia, 17 (74%, 17/23) entries were considered unique entries. In sum, only 2.2% (17/772) of our original 772 surveys included for analysis were deemed usable data at the conclusion of our analyses.

**Figure 2:**
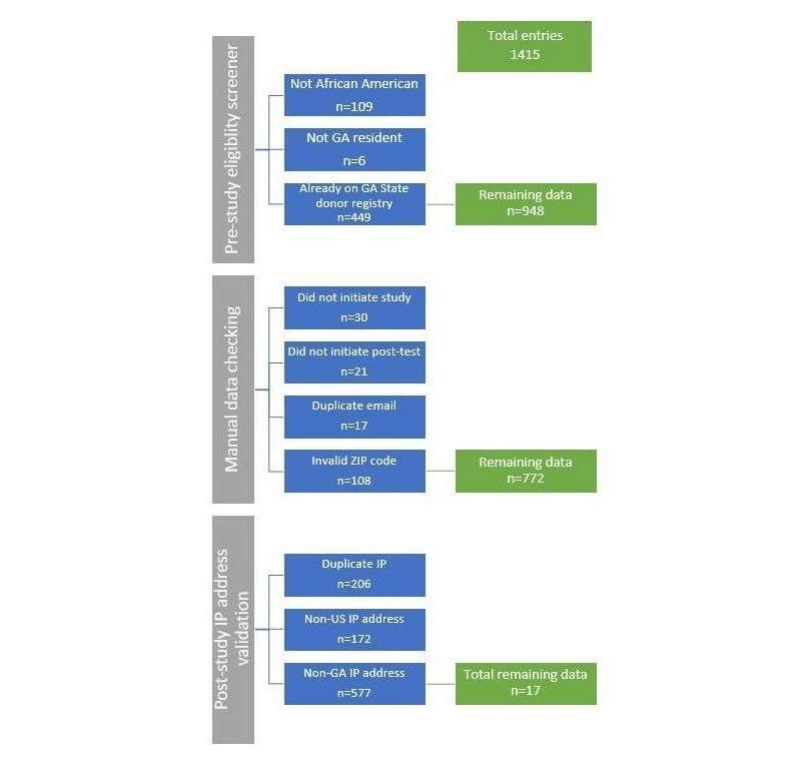
Summary of eliminated data through Reach assessment.

Although respondents with IP addresses outside of Georgia (United States and international) were not considered usable data, all IP addresses were analyzed to determine the extent of the threat of repeat responders to the entire study. Of the 577 international IP addresses, 207 (35.9%) completed the study more than one time. Of the 600 IP addresses within the United States, 144 (24.0%) completed the study more than one time. Chronic repeaters were seen among both US and international IP addresses, with respondents with international IPs presenting a more aggressive threat. Respondents with international IP addresses repeated up to 47 times, with the next highest repeats totaling 35 and 36 times. Respondents with US IPs repeated up to 35 times, with the next highest repeat being 18 times from one IP address (Figure 2; data points are not mutually exclusive).

### Recruitment

Approximately 517 participants of the 772 entries included after manual data cleaning (67.0%) reported that they were directly recruited from a trained Web recruiter, and the remainder from other means (eg, Craigslist website posting). Each Web recruiter recruited between 11 and 62 participants, with an average of 32 participants per recruiter.

**Table 2:** Participant demographics (N=772; sample sizes and percentages vary because of missing data).

Characteristic		Frequency
Age, mean (range)		36.16 (18-74)
**Gender, n (%)**		
	Female	332 (43.0)
	Male	434 (56.2)
**Education, n (%)**		
	Less than high school	17 (2.2)
	12th grade or GED	292 (37.8)
	College	382 (49.5)
	Professional degree	71 (9.2)
**Employment, n (%)**		
	Unemployed	15 (1.9)
	Retired	12 (1.6)
	Working part-time or full-time	728 (94.3)
**Marital status, n (%)**		
	Single	158 (20.5)
	Married	577 (74.7)
	Divorced or separated or widowed	17 (2.2)

### Dose Received

Website usage analytics showed that 1056 individuals accessed the study website home page (including intervention and control participants). Approximately 90.13% of these website visits were considered “new” or accessed by different individuals. A total of 417 participants were randomized to the intervention group; only two entries of the intervention group were among the 17 data entries deemed as valid from our reach analysis. Those in the intervention group who viewed the WEB ACTS website had an average session duration of 10 min 17 s, just 17 s over the required minimum. Activity on each of the WEB ACTS modules decreased as participants moved to the next module. The first module (Act Now), for example, had 538 page views (1.29 page views per user), and the average time spent on the page was about 6 min. The shift from the first module to the next module, Fairness in Organ Allocation, produced the greatefst drop-off in participation, with only 149 page views (0.36 page views per user) and an average page time of about 2 min. Participation continued to lessen with each subsequent module, with the final module, Let’s Talk About life, having only 68 page views (0.16 page views per user) but a slight peak in average viewing time (2:20 average page time).

Video viewing for each module was also minimal. Thirty-four participants pressed play for the Act Now video, 10 for the Fairness in Organ Allocation video, and 8 for the Fairness in Health Care Delivery video. The most of any video watched was the Fairness in Organ Allocation video; one person watched 70% of the video. No videos were watched in their entirety (Table 3).

### Context

Minimal study activity was observed during the first weeks the study was active. Responses to the eligibility screener averaged about 1.61 responses per day. Response to the study increased significantly once the Craigslist ad was placed; a total of 485 responses were recorded during the 24 hours that the ad was posted, and responses averaged 55.12 per day from the time the advertisement was removed until the study closed.

**Table 3:** WEB ACTS (About Choices in Transplantation and Sharing) website usage statistics (n=417).

Module^a^	Title	Number of page views (views per person)	Average time on page (min:sec)	Number of times pressed “play” for video	Highest percentage of video completed n (%)
Module I	Act Now	538 (1.29)	06:02	34	2 (50)
Module II	Fairness in Organ Allocation	149 (0.36)	02:08	10	1 (70)
Module III	Fairness in Health Care Delivery	108 (0.26)	01:28	8	1 (20)
Module IV	Religious Beliefs	104 (0.25)	01:25	11	1 (10)
Module V	Let’s Talk About Life	68 (0.16)	02:20	13	1 (50)

^a^Module videos were of different lengths and as follows: module I: 02:40, module II: 05:15, module III: 05:55, module IV: 05:43, and module V: 04:02.

The proportion of respondents found eligible increased the longer the study stayed active and particularly shifted at a certain time point of the study. Nearly 88.9% (431/485) of respondents on the first to the 28th day of the study were deemed ineligible; on the 29th day to day 60 (the end of the study), only 10.97% (102/930) of respondents were determined to be ineligible.

Web recruiters noted mostly using Facebook to recruit participants and said that they posted about the study on their personal accounts 3 to 4 times per day, on average. Some Web recruiters mentioned their Facebook friends reposting the study on their own pages, although we are not sure to what extent this occurred during the study. Overall, Web recruiters said they received minimal feedback about the study from friends on social media, but some mentioned being questioned whether the study was “real” or just spam.

## Discussion

### Principal Findings

This study sought to explore threats to Web-based interventions through the use of a process evaluation model refined for Internet settings. Whereas previous research suggests that fraud in Internet studies is minimal [35], we found fraudulent responses to be pervasive in our study, greatly impacting the overall quality of our data. Our study found that nearly 97.8% (755/772) of participants’ entries were invalid, despite having provided eligible responses on the eligibility screener. This finding supports the notion that ineligible and repeat participants are eager to infiltrate Web-based studies to receive monetary incentives, potentially creating a high volume of invalid responses. With a range of security parameters for Web-based studies available and no clear protocol on which strategies to use or to what extent to use them, assessing reach following our adapted process measures helped inform how to better secure our data collection protocol. In addition to using a CAPTCHA function to potentially protect the study from “bots,” we also learned that screening for IP addresses before study initiation is necessary to ensure study eligibility, particularly for studies requiring that participants reside in a particular geographical region.

Dose received analysis confirmed that uptake of the Web-based intervention was nominal, reaffirming findings regarding minimal uptake in prior studies [40,41]. Overall, website navigation for the intervention group was low, particularly as modules progressed. Additionally, no videos were watched in their entirety, and only 8% of participants pressed play for the first video, which was the most played of any video. Our initial study protocol implemented a minimal time requirement to remain on the website but did not mandate a minimal viewing time for website modules in the WEB ACTS site. This strategy was meant to encourage participants to explore modules in which they were most interested and that best fit their individually identified barriers to organ donation. And although we know that more uptake does not necessarily translate to more positive outcomes [32], given the negligible amount of uptake observed in our study, a minimum uptake requirement should be instituted to attribute any observed changes in outcomes to the intervention. It is important to note that the high volume of fraudulent respondents also potentially influenced minimal uptake of the intervention (ie, we would not expect video viewing to be high among fraudulent and repeat respondents who are seemingly accessing the study to receive multiple incentives). This interrelatedness of fraud and minimal uptake further underscores the importance of protecting Web-based studies from such threats.

Determining the optimal amount of study uptake to produce desired outcomes and prevent study attrition is difficult, as previous findings on what works varies [21,32,46], and to our knowledge, there are no published studies that outline how to determine this minimum. We suggest conducting efficacy testing before launching Web-based interventions to determine this optimal level of study consumption for specific study topics and demographics. In those cases where a mandate cannot be made because of technological limitations, knowing how much of an intervention individual participants consumed can be just as valuable, as these data would provide an opportunity to link consumption levels to outcomes [46]. Although we were only able to collect aggregate-level data to assess dose received, looking at individual consumption should be explored in future studies.

Context analysis provided us with valuable insights into factors in the Internet environment that may have affected study implementation. Although only active for a brief period of time, the Craigslist advertisement may have contributed the largest volume of fraudulent responses (our recruitment analysis determined that only 33.0% (255/772) of participants said they were recruited by the Craigslist ad; however, given the extent of fraudulent responses, these data were not considered to be reliable). We saw an exponential increase in responses to the eligibility screener after the Cragislist advertisement was posted, and most of those responses were determined to be fraudulent. We also observed that as time passed, more individuals were determined to be eligible for the study, suggesting that these fraudulent responders may have learned the desired eligibility and augmented answers on the screener to gain access to the study.

Conducting an assessment of context provided valuable information pertaining to program implementation, but such an assessment was difficult to conduct in an Internet environment, partially because of limited access to data on outside websites used to advertise our study (such as IP addresses of individuals accessing Craigslist or Facebook clicks). Despite these challenges, we found that using proxy data as an indicator to assess context provided valuable insights regarding how the Web-based study environment may have affected implementation and suggest using similar indicators when assessing context in Web-based studies. Steckler and Linnan [45] advise outlining potentially influential contextual factors before study initiation. For example, access to local recreational facilities would be an important contextual factor to consider if implementing a physical activity intervention, as it may be that participants with greater access to such facilities experience greater success with the intervention [45]. We also anticipate this practice would be valuable in Web-based study environments and may involve monitoring activity on websites associated with study marketing before implementation and throughout the entire study process, particularly when using social media sites that may involve interactions between individuals.

Although our context analysis provided some explanation as to how our study design may have facilitated fraudulent responses, the question of why so few legitimate participants were recruited remains. We learned from debriefing interviews with Web-based recruiters that even though some potential participants learned of the study from Web recruiters who they knew personally, there was still some skepticism around participating in the study. This suspicion may stem from concerns about compromising Internet privacy by clicking on foreign links, and/or interpreting unfamiliar links as “spam” [62], or may also stem from the topic of organ donation itself. As previously stated, organ donation is an altruistic health behavior that is difficult to change [42] and may elicit skepticism from African Americans, in particular, given overall feelings of distrust toward the American health care and organ allocation system [43]. And whereas research has found the Internet to be a feasible modality for implementing behavioral studies on sensitive topics [14-16,19,20], introducing organ donation on the Internet may heighten uncertainty around a controversial topic. More research is needed to determine how Web recruiters can be used to recruit participants into Web-based research studies using social media (particularly for sensitive topics) and how to make organ donation research participation more appealing to the general public.

### Limitations

There are several limitations to this study. First, portions of our process evaluation model were implemented retrospectively and not in tandem with study implementation, which would have been ideal to protect study data. For example, data regarding fraudulent participants through our reach analysis would have prompted us to implement tighter protocols mid-study, which could have led to much different overall results of the study. Second, some indicators used to measure our adapted process measures (such as data trends for context) were proxy measures and not direct measures of study implementation. And, whereas process evaluation indicators often rely on second-source feedback (such as interviews) [45], more vetting of these methods is needed to determine best practices for collecting this type of data via the Internet. Furthermore, because Web recruiter debriefing interviews were not required, only those recruiters who were interested provided feedback, allowing for some bias in the data collected. Finally, our adapted process evaluation model was only implemented on our study addressing organ donation behaviors among African Americans; more research is needed to see how this model can be used with Web-based interventions addressing other health topics among different populations.

### Conclusions

We found our process evaluation of the WEB ACTS study to be helpful in assessing the implementation of a culturally sensitive, Web-based educational intervention. Fraudulent responses and insufficient intervention uptake were identified as threats to our study. On the basis of our findings, we suggest checking participants’ IP addresses before study initiation, selecting software that allows for automatic duplicate protection, and tightening minimum requirements for intervention uptake. Whereas these parameters may have some trade-offs (including potentially deterring some legitimate participants), we anticipate these threats to be minimal and protecting the study to be of the highest priority. Clearly, implementing Web-based studies is complex and begs for continual monitoring through process evaluation models fit for Web-based settings. Further research is needed to understand how process evaluation models can be used to monitor implementation of Web-based studies.

## Abbreviations

ACTS: About Choices in Transplantation and Sharing
CAPTCHA: Completely Automated Public Turing test to tell Computers and Humans Apart
IP: Internet protocol

## References

[1] Ganikos M, Siegel JT, Alvaro EM (2010). Organ donation: an overview of the field. Understanding Organ Donation: Applied Behavioral Science Perspectives.

[2] U.S. Department of Health and Human Services (2015). Optn.transplant.hrsa.

[3] Anantachoti P, Gross CR, Gunderson S (2001). Promoting organ donation among high school students: an educational intervention. Prog Transplant.

[4] Birkimer JC, Barbee AP, Francis ML, Berry MM, Deuser PS, Pope JR (1994). Effects of refutational messages, thought provocation, and decision deadlines on signing to donate organs. J Appl Social Pyschol.

[5] Cantarovich F, Fagundes E, Biolcalti D, Bacqué MC (2000). School education, a basis for positive attitudes toward organ donation. Transplant Proc.

[6] Carducci BJ, Denser PS, Bauer A, Large M, Ramaekers M (1989). An application of the foot in the door technique to organ donation. J Bus Psychol.

[7] Kopfman JE, Smith SW (2009). Understanding the audiences of a health communication campaign: a discriminant analysis of potential organ donors based on intent to donate. J Appl Comm Res.

[8] Kopfman JE, Smith SW, Ah Yun JK, Hodges A (1998). Affective and cognitive reactions to narrative versus statistical evidence organ donation messages. J Appl Commun Res.

[9] Radecki CM, Jaccard J (1997). Psychological aspects of organ donation: a critical review and synthesis of individual and next-of-kin donation decisions. Health Psychol.

[10] Smith SW, Morrison K, Kopfman JE, Ford LA (1994). The influence of prior thought and intent on the memorability and persuasiveness of organ donation message strategies. Health Commun.

[11] Weaver M, Spigner C, Pineda M, Rabun KG, Allen MD (2000). Knowledge and opinions about organ donation among urban high school students: pilot test of a health education program. Clin Transplant.

[12] Pew Research Center (2017). Pewinternet.

[13] Portnoy DB, Scott-Sheldon LA, Johnson BT, Carey MP (2008). Computer-delivered interventions for health promotion and behavioral risk reduction: a meta-analysis of 75 randomized controlled trials, 1988-2007. Prev Med.

[14] Castillo-Arcos Ldel C, Benavides-Torres RA, López-Rosales F, Onofre-Rodríguez DJ, Valdez-Montero C, Maas-Góngora L (2016). The effect of an Internet-based intervention designed to reduce HIV/AIDS sexual risk among Mexican adolescents. AIDS Care.

[15] Marsch LA, Guarino H, Grabinski MJ, Syckes C, Dillingham ET, Xie H, Crosier BS (2015). Comparative effectiveness of web-based vs. educator-delivered HIV prevention for adolescent substance users: a randomized, controlled trial. J Subst Abuse Treat.

[16] Jones R, Hoover DR, Lacroix LJ (2013). A randomized controlled trial of soap opera videos streamed to smartphones to reduce risk of sexually transmitted human immunodeficiency virus (HIV) in young urban African American women. Nurs Outlook.

[17] O'Kearney R, Kang K, Christensen H, Griffiths K (2009). A controlled trial of a school-based Internet program for reducing depressive symptoms in adolescent girls. Depress Anxiety.

[18] Thompson NJ, Patel AH, Selwa LM, Stoll SC, Begley CE, Johnson EK, Fraser RT (2015). Expanding the efficacy of Project UPLIFT: Distance delivery of mindfulness-based depression prevention to people with epilepsy. J Consult Clin Psychol.

[19] Jones M, Taylor Lynch K, Kass AE, Burrows A, Williams J, Wilfley DE, Taylor CB (2014). Healthy weight regulation and eating disorder prevention in high school students: a universal and targeted Web-based intervention. J Med Internet Res.

[20] Heinicke BE, Paxton SJ, McLean SA, Wertheim EH (2007). Internet-delivered targeted group intervention for body dissatisfaction and disordered eating in adolescent girls: a randomized controlled trial. J Abnorm Child Psychol.

[21] Danaher BG, Seeley JR (2009). Methodological issues in research on web-based behavioral interventions. Ann Behav Med.

[22] Morgan AJ, Jorm AF, Mackinnon AJ (2013). Internet-based recruitment to a depression prevention intervention: lessons from the Mood Memos study. J Med Internet Res.

[23] Frandsen M, Walters J, Ferguson SG (2014). Exploring the viability of using online social media advertising as a recruitment method for smoking cessation clinical trials. Nicotine Tob Res.

[24] Yuan P, Bare MG, Johnson MO, Saberi P (2014). Using online social media for recruitment of human immunodeficiency virus-positive participants: a cross-sectional survey. J Med Internet Res.

[25] Lohse B (2013). Facebook is an effective strategy to recruit low-income women to online nutrition education. J Nutr Educ Behav.

[26] Gordon JS, Akers L, Severson HH, Danaher BG, Boles SM (2006). Successful participant recruitment strategies for an online smokeless tobacco cessation program. Nicotine Tob Res.

[27] Watson B, Robinson DH, Harker L, Arriola KR (2016). The inclusion of African-American study participants in web-based research studies: viewpoint. J Med Internet Res.

[28] Williamson DA, Martin PD, White MA, Newton R, Walden H, York-Crowe E, Alfonso A, Gordon S, Ryan D (2005). Efficacy of an internet-based behavioral weight loss program for overweight adolescent African-American girls. Eat Weight Disord.

[29] Vinokur AD, Merion RM, Couper MP, Jones EG, Dong Y (2006). Educational web-based intervention for high school students to increase knowledge and promote positive attitudes toward organ donation. Health Educ Behav.

[30] Merion RM, Vinokur AD, Couper MP, Jones EG, Dong Y, Wimsatt M, Warren J, Katz S, Leichtman AB, Beyersdorf T (2003). Internet-based intervention to promote organ donor registry participation and family notification. Transplantation.

[31] Lockwood MB, Saunders MR, Lee CS, Becker YT, Josephson MA, Chon WJ (2013). Kidney transplant and the digital divide: is information and communication technology a barrier or a bridge to transplant for African Americans?. Prog Transplant.

[32] Glasgow RE, Nelson CC, Kearney KA, Reid R, Ritzwoller DP, Strecher VJ, Couper MP, Green B, Wildenhaus K (2007). Reach, engagement, and retention in an Internet-based weight loss program in a multi-site randomized controlled trial. J Med Internet Res.

[33] Bauermeister JA, Pingel E, Zimmerman M, Couper M, Carballo-Dieguez A, Strecher VJ (2012). Data quality in HIV/AIDS web-based surveys: handling invalid and suspicious data. Field Methods.

[34] Gosling SD, Vazire S, Srivastava S, John OP (2004). Should we trust web-based studies? A comparative analysis of six preconceptions about internet questionnaires. Am Psychol.

[35] Teitcher JE, Bockting WO, Bauermeister JA, Hoefer CJ, Miner MH, Klitzman RL (2015). Detecting, preventing, and responding to “fraudsters” in internet research: ethics and tradeoffs. J Law Med Ethics.

[36] Konstan JA, Simon Rosser BR, Ross MW, Stanton J, Edwards WM (2005). The story of subject naught: a cautionary but optimistic tale of Internet survey research. J Comput Mediat Commun.

[37] Grey JA, Konstan J, Iantaffi A, Wilkerson JM, Galos D, Rosser BRS (2015). An updated protocol to detect invalid entries in an online survey of men who have sex with men (MSM): how do valid and invalid submissions compare?. AIDS Behav.

[38] Mustanski BS (2001). Getting wired: exploiting the Internet for the collection of valid sexuality data. J Sex Res.

[39] Bowen AM, Daniel CM, Williams ML, Baird GL (2008). Identifying multiple submissions in Internet research: preserving data integrity. AIDS Behav.

[40] Brouwer W, Kroeze W, Crutzen R, de Nooijer J, de Vries NK, Brug J, Oenema A (2011). Which intervention characteristics are related to more exposure to internet-delivered healthy lifestyle promotion interventions? A systematic review. J Med Internet Res.

[41] Moltu C, Stefansen J, Svisdahl M, Veseth M (2012). Negotiating the coresearcher mandate - service users' experiences of doing collaborative research on mental health. Disabil Rehabil.

[42] Siegel JT, Alvaro EM, Hohman ZP (2010). A dawning recognition of factors for increasing donor registration: The IIFF Model. Understanding Organ Donation: Applied Behavioral Science Perspectives.

[43] Kurz RS, Scharff DP, Terry T, Alexander S, Waterman A (2007). Factors influencing organ donation decisions by African Americans: a review of the literature. Med Care Res Rev.

[44] Pequegnat W, Rosser BR, Bowen AM, Bull SS, DiClemente RJ, Bockting WO, Elford J, Fishbein M, Gurak L, Horvath K, Konstan J, Noar SM, Ross MW, Sherr L, Spiegel D, Zimmerman R (2007). Conducting Internet-based HIV/STD prevention survey research: considerations in design and evaluation. AIDS Behav.

[45] Steckler A, Linnan L (2002). Process evaluation for public health interventions and research: an overview. Process Evaluation for Public Health Interventions and Research.

[46] Christensen H, Griffiths KM, Korten A (2002). Web-based cognitive behavior therapy: analysis of site usage and changes in depression and anxiety scores. J Med Internet Res.

[47] Cummins CO, Prochaska JO, Driskell M, Evers KE, Wright JA, Prochaska JM, Velicer WF (2003). Development of review criteria to evaluate health behavior change websites. J Health Psychol.

[48] O'Grady L, Witteman H, Bender JL, Urowitz S, Wiljer D, Jadad AR (2009). Measuring the impact of a moving target: towards a dynamic framework for evaluating collaborative adaptive interactive technologies. J Med Internet Res.

[49] van Gemert-Pijnen JE, Nijland N, van Limburg M, Ossebaard HC, Kelders SM, Eysenbach G, Seydel ER (2011). A holistic framework to improve the uptake and impact of eHealth technologies. J Med Internet Res.

[50] Evers KE (2006). eHealth promotion: the use of the Internet for health promotion. Am J Health Promot.

[51] Bock B, Graham A, Sciamanna C, Krishnamoorthy J, Whiteley J, Carmona-Barros R, Niaura R, Abrams D (2004). Smoking cessation treatment on the Internet: content, quality, and usability. Nicotine Tob Res.

[52] Danaher BG, Smolkowski K, Seeley JR, Severson HH (2008). Mediators of a successful web-based smokeless tobacco cessation program. Addiction.

[53] Escoffery C, McCormick L, Bateman K (2004). Development and process evaluation of a web-based smoking cessation program for college smokers: innovative tool for education. Patient Educ Couns.

[54] Saunders RP, Evans MH, Joshi P (2005). Developing a process-evaluation plan for assessing health promotion program implementation: a how-to guide. Health Promot Pract.

[55] Resnicow K, Baranowski T, Ahluwalia JS, Braithwaite RL (1999). Cultural sensitivity in public health: defined and demystified. Ethn Dis.

[56] Callender CO, Miles PV (2001). Obstacles to organ donation in ethnic minorities. Pediatr Transplant.

[57] Callender CO, Miles PV (2010). Minority organ donation: the power of an educated community. J Am Coll Surg.

[58] Arriola K, Robinson DH, Thompson NJ, Perryman JP (2010). Project ACTS: an intervention to increase organ and tissue donation intentions among African Americans. Health Educ Behav.

[59] Arriola KR, Robinson DH, Perryman JP, Thompson NJ, Russell EF (2013). Project ACTS II: organ donation education for African American adults. Ethn Dis.

[60] Arriola KR, Powell CL, Thompson NJ, Perryman JP, Basu M (2014). Living donor transplant education for African American patients with end-stage renal disease. Prog Transplant.

[61] Thorogood M, Coombes Y (2010). Evaluating Health Promotion: Practice and Methods.

[62] Shannon DM, Johnson TE, Searcy S, Lott A (2002). Using electronic surveys: advice from survey professionals. Pract Assess Res Eval.

